# Editorial: 50 years of BMT: risk stratification, donor matching and stem cell collection for transplantation

**DOI:** 10.3389/fonc.2023.1321334

**Published:** 2023-12-19

**Authors:** Nelson Hamerschlak, David Gómez-Almaguer, Donal P. McLornan

**Affiliations:** ^1^ Hospital Israelita Albert Einstein, Department of Bone Marrow Transplant, São Paulo, SP, Brazil; ^2^ Servicio de Hematología, Hospital Universitario, Universidad Autánoma de Nuevo León, Monterrey, Mexico; ^3^ Department of Haematology and Stem Cell Transplantation, University College London Hospitals NHS Trust, London, United Kingdom

**Keywords:** BMT, medical history, stem cells, CAR-T, CAR-T cells

Hematopoietic stem cell transplantation (HSCT) is a treatment recommended for transplant-eligible patients with non-functioning bone marrow, certain haematological malignancies, and specific genetic and autoimmune diseases (1). HSCT has been used for more than six decades. In 1957, E. Donnall Thomas and colleagues reported on patients with advanced leukaemia who received marrow-derived haematopoietic stem cells following treatment with a high dose of total body irradiation (TBI) (2). This first report of successful allogeneic HSCT showed that ablative TBI followed by infusion of compatible haematopoietic stem cells was feasible.

The concept is based on the infusion of hematopoietic stem cells (HSC) from the patient (autologous) or from a donor (allogeneic). Once selected as a transplant candidate following careful evaluation, the patient receives high-dose chemotherapy and/or radiotherapy in order to provide ‘physical space’ within the marrow, with immunosuppression and eradication or reduction of the underlying disease.

As reported by Bortin, of the 200 patients treated between the decade spanning 1957 through to 1967, none of them, unfortunately, had long-term survival (3). However, the scientific creativity and prowess of the pioneers in the field changed the fate of this complex procedure. Improvements in human leucocyte antigen (HLA)-typing, different preparative regimens adjusted for disease-risk and recipient age/co-morbidities, graft-versus-host disease (GVHD) prophylaxis, and improved care and treatment before and after the transplant made it possible to reach the milestone of 1.5 million HSCTs performed worldwide between 1957 to 2019 (4).

But what has changed in these 50 years? In fact, everything.

Let’s start with the most important aspect: human resources. The transplant process gradually required new professionals to optimise the procedure, paralleled with future-thinking strategies. Today we know that it is essential to have a good team of clinicians from different specialties alongside stem cell laboratory personnel (5). We moved to selection of new sources of HSC from peripheral and umbilical cord blood (6). More options are hence available for patients, especially those with no related donors. Currently, the vast majority of patients will have a suitable donor (7–9).

This edition of Frontiers in Oncology presents a systematic review with meta-analysis concerning outcomes with mismatched unrelated donor (MMUD) allogeneic HSCT in adults. The review included data from 19 studies involving 3,336 patients who underwent MMUD-HSCT. Most recipients received peripheral blood stem cells (81%), and a significant proportion had reduced intensity conditioning (RIC; 65.6%). The combined overall survival (OS) at one year was 63.9%, while the estimate at three years was 42.1%. Acute GVHD of grades II–IV occurred in 36.4% of the cases, while chronic GVHD was 41.2%. Non-relapse mortality was observed in 22.6%. In conclusion, MMUD-HSCT presents a promising alternative for patients lacking a HLA-matched donor or a readily available haploidentical donor, broadening the availability of HSCT. The question in the field is whether to choose a MMUD or haploidentical donor in many disease types, and ongoing studies are evaluating this question, in particular regard to both OS and GVHD-free/relapse-free survival (GRFS) (10, 11). Use of risk-adapted scores gives greater information and potentiates earlier intervention in high-risk cases. We should also be aware of the emerging data on the use of post-transplant cyclophosphamide (PTCy) in this setting, with some studies suggesting lower rates of both acute and chronic GVHD (12–14).

Over the past six decades, we began to improve our understanding of HLA compatibility at a much higher resolution, which facilitated improved matching capability. We have new information regarding the DP permissive/non-permissive locus, KIR match, HLA B leader, HLA loss, and anti-donor specific antibodies. Therefore, rejection and GVHD rates have decreased (7, 15).

We developed new ways for evaluating patients, for example, increasing the age of those eligible for transplantation through comprehensive geriatric assessment (16). The hematopoietic cell transplantation-specific comorbidity index (HCT-CI) is a useful tool for risk assessment of comorbidities before allogeneic HSCT that can aid estimation of non-relapse mortality and survival (17).

International patient registries have been developed, facilitating many studies in collaboration, such as the CIBMTR (https://cibmtr.org) and EBMT (https://www.ebmt.org). Donor registries such as NMDP, DKMS, and REDOME also expand the availability of donors for patients (18). Brazil has the third most established registry globally, with more than seven million donors. International collaborations facilitate multicentric clinical trials conducted to optimise transplant platforms and outcomes (19). For example, we recently published results of a phase I study in high-risk acute myeloid leukaemia (AML) patients showing the benefit of total marrow irradiation combined with chemotherapy (20). Increasing the use of reduced intensity and non-myeloablative regimens expands the transplant option to older individuals or those with co-morbidities. However, more intense conditioning regimens may still benefit younger patients (21).

We expanded the recommendations for transplantation to include hemoglobinopathies and autoimmune diseases. However, in cases such as chronic myeloid leukaemia, we stopped performing transplants and began giving patients “one pill a day” (22–24). We also learned from haplo transplants using PTCy as discussed above (25).

Reducing the risk of relapse post-transplant is a big challenge. In this edition, a study evaluates isolated central nervous system acute lymphoblastic leukaemia (CNS-ALL) prior to allo-HCT using flow cytometry (FC) before allo-HSCT in a large patient cohort (n=1406) with ALL in complete remission (CR). Patients were grouped into three categories: isolated CSM involvement in flow cytometry (FCM) cytology-positive CNS involvement, and negative CNS involvement. The five-year cumulative incidence of relapse (CIR) was significantly higher in the isolated FCM-positive group (42.3%) and cytology-positive group (48.8%) compared to the negative CNS involvement group (23.4%). Multivariate analysis identified four risk factors associated with higher CR and inferior leukaemia-free survival (LFS): T-cell ALL, being in second CR or beyond at time of allo-HSCT, pre-HSCT measurable residual disease (MRD) positivity, and pre-HSCT CNS involvement. A novel risk scoring system was developed, categorising patients into low-risk, intermediate-risk, high-risk, and extremely high-risk groups. The five-year CIR and LFS rates varied significantly, with extremely high-risk patients having the highest CIR (66.7%) and the lowest LFS (13.3%). In conclusion, as expected, patients with isolated FCM-positive CNS involvement before transplant are at a higher risk of leukaemia recurrence post allo-HSCT. Prospective studies of novel agents and adaptive transplant platforms are urgently needed in this field to minimise relapse risk and improve longer term survival.

Considerable advances in both treatment and prevention of infections have been made. The advent of new tools and protocols to detect and treat fungal infections improved control of infections by *Aspergillus*, mucormycosis, and *Candida*. The early diagnosis and prevention of *Cytomegalovirus* were considerable advances, as well as treatment of reactivation/persistence (26). New drugs and procedures have emerged to prevent and treat major transplantation complications such as mucositis, hepatic veno-occlusive disease, and GVHD. Bone marrow transplant (BMT), hence, has become safer (27). Measurable residual disease (MRD) status detected by multiparameter flow cytometry or molecular techniques is of key importance in ALL and AML. Strong evidence suggests that MRD status should be used for risk stratification in acute leukaemia at the time of HSCT. Even in multiple myeloma, MRD has been an excellent marker of progression-free survival (28).

In this edition, there is a study focused on paediatric patients diagnosed with acute megakaryoblastic leukaemia (AMKL) but without Down syndrome (DS), an uncommon yet highly aggressive disorder. The study evaluated the outcomes of haploidentical-HSCT in 25 AMKL patients who underwent haploidentical HSCT. The two-year OS and event-free survival (EFS) were 54.5 (± 10.3) % and 50.9 (± 10.2)%, respectively. Notably, patients with trisomy 19 had a statistically significantly better EFS (80 (± 12.6)% versus 33.3 (± 12.2%). The two-year CIR was 46.1 (± 11.6)%, and one patient died post HSCT due to respiratory complications. In summary, paediatric AMKL without DS represents a rare and highly aggressive disease. This study confirms that haploidentical HSCT might be a viable option for high-risk paediatric AMKL without DS.

All advances in HSCT practice have resulted in a reduction in non-relapse mortality. Unfortunately, relapse after transplant is still the main mortality cause in our patients. Strategies to prevent and treat relapse have been reported (28, 29).

Bone marrow transplant units are now rapidly expanding into the cellular therapy arena as car-T, NK cells, adoptive anti-virus specific T cells, and mesenchymal cells for GVHD are increasingly being used (30–32).

An overview of the last 50 years in HSCT is presented in this Research Topic. The articles offer a contemporary and practical overview of recipient risk index with a focus on comorbidities, remission status, and cytogenetics prior to allo-HSCT. The authors also suggest how best to make stem cell source choices and improve stem cell collection efficiency. The experience of the authors and a literature overview make this contribution a very practical guide for practice optimisation in HSCT centres.

All articles in this edition provide more data towards the goal of improving accessibility and outcomes for patients requiring HSCT globally. Enjoy the reading!

## Author contributions

NH: Conceptualization, Investigation, Writing – original draft, Writing – review & editing. DG-A: Investigation, Writing – review & editing. DM: Conceptualization, Investigation, Writing – review & editing.
